# How does the UK public think and feel about people with visual impairment: a review of existing evidence

**DOI:** 10.3389/fpsyg.2024.1359074

**Published:** 2024-03-07

**Authors:** Nikki Heinze, Lee Jones, Firuzé Bertiz, Emma Saunders, Renata S. M. Gomes

**Affiliations:** ^1^BRAVO VICTOR, London, United Kingdom; ^2^Institute of Ophthalmology, University College London, London, United Kingdom; ^3^Royal National Institute of Blind People, London, United Kingdom; ^4^Northern Hub for Veterans and Military Families Research, Department of Nursing, Midwifery and Health, Faculty of Health and Life Sciences, Northumbria University, Newcastle upon Tyne, United Kingdom

**Keywords:** visual impairment, sight loss, public attitudes, perceptions, behaviour

## Abstract

Despite legislation to protect people with visual impairment (V.I.) from discrimination in the United Kingdom (UK), the latter continue to experience overt and covert negative behaviours. Perceived discrimination has been associated with an adverse impact on identity, health and well-being, while negative attitudes have been identified as the biggest barrier to participation in everyday life. This article provides a narrative review of existing evidence of how the UK public treats (behaviours), thinks (perceptions) and feels (attitudes) about people with V.I. Despite limitations, the findings suggest that there is a gap between the behaviours reported by people with V.I. and the attitudes expressed by members of the UK public. Social psychological theories are used to explore possible reasons for this gap, and ways in which it may be addressed. As such, the article provides an example of how social psychological theories can be used to address problems in an applied context.

## 1 Introduction

In the United Kingdom (UK), the number of people living with visual impairment (V.I.) is estimated to increase from ~2 million to ~4 million by 2050 (Pezzullo et al., 2018). Definitions of V.I. vary, but generally refer to impaired sight in one or both eyes which cannot be corrected with glasses or contact lenses and can range from partial sight to total blindness with no light perception. V.I. can be congenital (present from birth) or acquired later in life, and affect central or peripheral vision or a combination, depending on the underlying eye condition or conditions.

Although there is considerable resilience in people living with disabilities (Shakespeare, 2013), V.I. has been associated with a negative impact on quality of life (QoL), psychosocial well-being, sleep quality and mental health (Tabandeh et al., 1998; Fenwick et al., 2012; Kempen et al., 2012; Nyman et al., 2012; Zhang et al., 2013; van der Aa et al., 2015; Cumberland and Rahi, 2016; Schliermann et al., 2017; Frank et al., 2019), though mental health outcomes may be poorer in those with acquired than congenital V.I. (Choi et al., 2019). In addition, V.I. has been associated with an adverse impact on activities of daily living such as shopping, self-care and household chores (Alma, 2011; Gopinath et al., 2014), participation in sports and leisure activities (Jaarsma et al., 2014; Phoenix et al., 2015) and employment outcomes (Coffey et al., 2014; Cumberland and Rahi, 2016). These adverse experiences may in part be the result of external factors such as barriers to transport use, inaccessible environments, and discriminatory attitudes and behaviours toward individuals with V.I. (Phoenix et al., 2015; Jin et al., 2019).

Negative experiences can have wide-ranging impacts. A 2022 UK survey of 4,015 adults with disabilities found that around a third avoided education (30%) and job opportunities (new jobs and promotions) (35%), a quarter avoided socialising (23%) and using public transport (23%), and around one in ten avoided health and social care (13%) due to negative experiences and attitudes (Moss and Frounks, 2022). Overall, 87% reported a negative impact of attitudes and behaviours on their lives (Moss and Frounks, 2022). This proportion was higher among younger adults and women.

Among people with V.I., perceived discrimination has been associated with a significant negative impact on a range of physical and mental health outcomes (Pascoe and Smart Richman, 2009). Findings from the English Longitudinal Study of Ageing (ELSA) showed that older adults with self-reported poor eyesight who had experienced discrimination were at increased risk of depression, loneliness, poorer life satisfaction and poorer QoL than those who had not experienced discrimination (Jackson et al., 2019). Perceptions and attitudes can also impact on a person’s sense of independence and belonging (Dale, 2010), confidence (RNIB, Guide Dogs, and TPT, 2022), and personal and social identities (Sanders, 2000). For instance, individuals with partial sight have described ‘feeling like a fraud’ because they have residual vision, which challenges public perceptions that typically equate V.I. with total blindness without light perception (Dale, 2010; Nyman et al., 2012). Research with adolescents found that, in addition to the perceived limitations imposed by their V.I. on meaningful activities such as driving or playing a musical instrument, discriminatory behaviours at school and from their peers resulted in social isolation (Rosenblum, 2000). While some viewed their V.I. as an integral part of their identity and indeed a way to break the ice when interacting with others, most reported disliking their V.I., feeling anger and frustration towards it, regardless of age of onset (Rosenblum, 2000).

By comparison, developing a positive disability identity has been associated with greater life satisfaction (Bogart, 2014) and a greater sense of belonging, which, in turn, may be linked to better physical health and mood, although the impact may be mediated by self-esteem (Begen and Turner-Cobb, 2012). Indeed, acceptance of V.I. has been linked with better well-being and lower levels of depression (Bergeron and Wanet-Defalque, 2013). This may be explained by Social identity theory (Tajfel et al., 1979) which allows for both benefits and disadvantages associated with a social identity. Benefits arise from intragroup processes including having a sense of identity, community and belonging to a social group. Disadvantages arise from intergroup processes which involve social comparison and intergroup bias. Strategies to deal with stigmatised social identities, such as those associated with disabilities, include social mobility, creativity and competition (Dirth and Branscombe, 2018). Social mobility involves attempting to become a member of a higher status group, which may be achieved by ‘passing’ or fitting in with the majority outgroup by concealing the disability and adopting the negative attitudes of the outgroup (Dirth and Branscombe, 2018; van Bezouw et al., 2021). However, concealing a V.I. in the context of employment is associated with increased stress and lower self-acceptance, while greater transparency and active steps to plan for deteriorating vision were associated with better well-being and self-acceptance (De Bel et al., 2016). Moreover, a reluctance to self-identify as visually impaired may have wider impacts on the use of essential eye health and support services for treatment, vision rehabilitation and emotional support to enable people to live well with V.I., and the acceptability of mobility aids such as guide dogs and canes which may signal V.I. to other people (Sanders, 2000; Nyman et al., 2012; Hersh, 2015). Anecdotes from qualitative research suggest that visual symbols of V.I., such as canes and guide dogs, can result in a more positive reaction and patience, thus improving interaction experiences. However, among those who are still coming to terms with their V.I., they may create a feeling of ‘otherness’ (RNIB, Guide Dogs, and TPT, 2022). Social creativity involves changing or reframing the negative value associated with an identity, for instance, by reappropriating terms used derogatorily or reinterpreting a negative social identity (Dirth and Branscombe, 2018; van Bezouw et al., 2021). In the context of V.I. this may involve reframing conversations from V.I. being a disabling condition of the body to it being a disabling condition due to social barriers or talking about different ‘capabilities’ rather than ‘disabilities’. Finally, social competition involves an active effort to change the unequal status of a stigmatised group and intergroup conflict (Dirth and Branscombe, 2018; van Bezouw et al., 2021). Examples include the disability rights movement, actively challenging discrimination and protesting for change. Survey research found that adopting a social model of disability and campaigning for disability rights were associated with a positive disability identity including disability pride and a sense of purpose (Grewal et al., 2002). Yet, some people with V.I. may feel a reluctant responsibility to become a positive representative for the collective of people with V.I. (“I do not want people to think all blind people are miserable, crummy people and all this other stuff, but it does get very frustrating.,” Sanders, 2000, p.135).

This highlights the potential impact of perceived negative behaviours, attitudes and perceptions on mental and physical health, identity, use of health and support services, and participation in everyday life. Indeed, people with V.I. have identified negative attitudes as one of the biggest barriers to their ability to participate in everyday life (Fiske et al., 2002). The following sections demonstrate that, while there is a body of academic and grey literature on perceived discrimination and attitudes, relatively little research has explored behaviours, attitudes and perceptions from the perspective of the UK public even though research in the context of disability (Dixon et al., 2018) and racism (Carter and Murphy, 2015) indicates that there may be a gap in perceived and espoused perceptions and attitudes towards a group. This narrative review provides a synthesis of existing evidence relating to how the UK public treat (behaviour), feel (attitudes) and think (perceptions) about people with V.I. This includes a review of how the UK public feel and think about V.I. itself to provide context for attitudes and perceptions towards those living with V.I. The article will then explore how social psychological theories can be used to make sense of the gap between perceived and espoused attitudes and experiences.

## 2 Methods

This narrative review provides an overview of evidence from the academic and grey literature. A search of charity websites and online databases was conducted using search terms relating to ‘*visual impairment*’ and ‘*public attitudes*’ (e.g., public perceptions, social attitudes, etc.). Due to the changing nature of attitudes, the focus was on research published since 2000. The “values, ideas and practices” (Moscovici, 1973, p. xiii) of a group are highly specific to their contexts (Moscovici, 1988). Considering cultural, legal and structural differences across different contexts, this article therefore focuses on behaviours, attitudes and perceptions in the UK. Due to the relative scarcity of existing evidence on behaviours, attitudes and perceptions of people with V.I., articles which did not meet the criteria were at times included in the review if they provided valuable insights. As such, a small number of articles relate to people with disabilities in general (e.g., Grewal et al., 2002), from outside the UK (e.g., Shpigelman and Vorobioff, 2019) and prior to 2000 (e.g., Fichten et al., 1991). Table 1 provides an overview of the articles and reports included in the following sections.

**Table 1:** Overview of articles and reports included in the narrative review.

**Paper**	**Source**	**Location**	**Methodology**	**Sample**	**Section**
Grewal et al. (2002)	Grey literature	GB	Mixed methods: Qualitative (interviews, focus groups) Quantitative (survey)	Qualitative: 150 adults aged 18+ (75 with disabilities) Quantitative: 2,064 adults aged 16+ (965 with disabilities)	Behaviours Attitudes and perceptions of V.I.
Aiden and McCarthy (2014)	Grey literature	Survey 1: UK Survey 2: GB	Surveys	Survey 1: 1,014 adults aged 18+ with disabilities Survey 2: 2,045 adults aged 16+ with disabilities	Behaviours
RNIB, Guide Dogs, and TPT (2022)	Grey literature	UK	Survey	769 blind and partially sighted people aged 13+	Behaviours Attitudes and perceptions of people with V.I.
McManus and Lord (2012)	Grey literature	GB	Secondary analysis of USoc and LOS data	USoC: 100,000 people in 40,000 households LOS: 37,500 households interviewing everyone in the household Ages 16+	Behaviours Attitudes and perceptions of people with V.I.
Flynn and Lord (2015)	Grey literature	GB	Secondary analysis of USoc and LOS data	Not specified	Behaviours
Jackson et al. (2019)	Academic literature	England	Secondary analysis of ELSA survey data	7,677 adults aged ≥50 (*n =* 913 with poor self-reported eyesight, *n* = 658 with poor eyesight up close)	Behaviours
Slade and Edwards (2015)	Grey literature	UK	Survey	1,223 blind and partially sighted adults aged 18+	Behaviours Attitudes and perceptions of people with V.I.
Heinze and Castle (2023)	Academic literature	UK	Secondary analysis of V I Lives survey data	154 adults aged 18+ with V.I. (*n* = 77 from minority ethnic communities)	Behaviours
Sanders (2000)	Academic literature	Northeast USA	Ethnographic fieldwork	6 guide dog trainers, 7 guide dog owners	Behaviours Attitudes and perceptions of V.I.
Slade et al. (2017)	Grey literature	UK, GB	Secondary analysis of My Voice, Network 1,000 and LFS data	>1,200 (My Voice), nearly 1,000 (Network 1,000) blind and partially sighted people	Behaviours
McDonnall and Lund (2020)	Academic literature	USA	Survey	388 hiring managers	Behaviours
Versiti (2018)	Grey literature	GB, UK	Mixed methods: Phase I: 24 daily research activities over 10 days (e.g., discussions, projective techniques, surveys, video tasks) Phase II: Implicit and explicit association tests	Phase I: 42 people Phase II: 1,000 people *Sample age was not specified*	Attitudes and perceptions of V.I. Attitudes and perceptions of people with V.I.
Biddyr et al. (2016)	Grey literature	Wales	Focus groups	23 adults (5 from Somali, 6 from African-Caribbean and 12 from Gujarati communities)	Attitudes and perceptions of V.I.
Patel et al. (2006)	Academic literature	England (West London)	Focus groups, interviews	44 community members, religious leaders, patients and health providers from the Indian community	Attitudes and perceptions of V.I.
Robinson et al. (2007)	Grey literature	GB	Secondary analysis of BSA survey data	3,193 adults aged 18+	Attitudes and perceptions of V.I. Attitudes and perceptions of people with V.I.
Enoch et al. (2019)	Academic literature	UK	Survey	250 adults aged 22+	Attitudes and perceptions of V.I.
Enoch et al. (2020)	Academic literature	UK	Survey	250 adults aged 22+	Attitudes and perceptions of V.I.
Hutmacher (2019)	Academic literature	N/A	Conceptual analysis	N/A	Attitudes and perceptions of V.I.
Moradi et al. (2007)	Academic literature	Bournemouth, Winchester, Manchester, Southampton, England	Survey	260 teenagers aged 16–18 years	Attitudes and perceptions of V.I.
Bidwell et al. (2005)	Academic literature	Bolton, England	Survey	358 adult patients aged 18+	Attitudes and perceptions of V.I.
Fisher et al. (2018)	Grey literature	UK	Choice based exercise Survey	656 adults aged 18+	Attitudes and perceptions of V.I.
Shickle and Griffin (2014)	Academic literature	Leeds, England	Focus groups	81 adults aged 31+	Attitudes and perceptions of V.I.
Shickle et al. (2014)	Academic literature	Leeds, England	Focus groups	43 adults aged 18–35	Attitudes and perceptions of V.I.
Dale (2010)	Academic literature	UK	Audio narrative	4 people with V.I.	Attitudes and perceptions of V.I.
Nyman et al. (2012)	Academic literature	N/A	Meta-synthesis of qualitative research	N/A	Attitudes and perceptions of V.I.
Cross et al. (2005)	Academic literature	Birmingham, England	Interview, focus groups	48 Afro-Caribbean participants	Attitudes and perceptions of V.I.
Cant and Bennett (2022)	Grey literature	GB	Analysis of BSA data	6,250 adults aged 18+	Attitudes and perceptions of people with V.I.
Moss and Frounks (2022)	Grey literature	UK	Survey	4,015 adults with different disabilities	Attitudes and perceptions of people with V.I.
Milligan and Neufeldt (2001)	Academic literature	N/A	Narrative review	N/A	Attitudes and perceptions of people with V.I. Asexuality
Kim (2011)	Academic literature	N/A	Essay	N/A	Attitudes and perceptions of people with V.I. Asexuality
Fichten et al. (1991)	Academic literature	Canada	Experiment, survey scales	330 college students aged 17–36 without disabilities	Attitudes and perceptions of people with V.I.
Shpigelman and Vorobioff (2019)	Academic literature	Israel	Interviews	24 Jewish and Arab adults aged 18–40	Attitudes and perceptions of people with V.I.
Nikolaraizi and De Reybekiel (2001)	Academic literature	West Midlands, England, Greece	Survey	234 UK and 229 Greek children aged 10–12 attending primary schools	Attitudes and perceptions of people with V.I.

USoc = Understanding Society, LOS = Life Opportunities Survey, ELSA = English Longitudinal Study of Ageing, LFS = Labour Force Survey, BSA = British Social Attitudes.

## 3 Behaviour – how the public treat people with V.I.

With the introduction of the Disability Discrimination Act in 1995 and the Equality Act 2010, it is illegal in the UK to discriminate against a person because of their disability. According to the Equality Act 2010, discrimination is defined as “treating someone less favourably than someone else because of a protected characteristic” (Equality and Human Rights Commission, 2019, p. 3). Protected characteristics according to the Act include age, disability, gender reassignment, marriage and civil partnership, pregnancy and maternity, race, religion and belief, and sex and sexual orientation (Equality and Human Rights Commission, 2019). The Act distinguishes between different types of discrimination, including direct discrimination, direct discrimination by perception or association, harassment, and victimisation. In the context of disability, discrimination also includes failing to make reasonable adjustments and treating someone with a disability “unfavourably because of something connected with their disability when this cannot be objectively justified” (Equality and Human Rights Commission, 2019, p. 5); for instance, by dismissing an employee because of disability-related sick leave. In addition to direct or overt discrimination, discrimination can also be indirect or covert. Indirect discrimination, according to the Act, relates to “putting in place a rule or policy or way of doing things that has a worse impact on someone with a protected characteristic than someone without one, when this cannot be objectively justified” (Equality and Human Rights Commission, 2019, p. 4).

This may not necessarily correspond with lay understandings of what constitutes discriminatory attitudes or behaviours. Indeed, people with disabilities continue to report prejudice, discrimination and social exclusion (Grewal et al., 2002). Findings from two UK surveys give an indication of the types and prevalence of behaviours experienced by people with disabilities (Aiden and McCarthy, 2014). These included not being believed that they had a disability (49%), being talked to in a patronising way (35%), being stared at (30%), people refusing to make reasonable adjustments or do things differently (28%), people incorrectly assuming that the respondent did not work because of their disability (21%), being called names (17%), people acting in an aggressive or hostile manner (16%) and being physically attacked (4%) (*n* = 1,014 UK adults aged 18 and over whose day-to-day activities are affected by long-term physical or mental impairments, conditions, illnesses or disabilities). The behaviours and attitudes people with disability had experienced and would most like to change were: people not understanding their needs (41% experienced/40% wanted change), being treated like a nuisance (23%/20%), people expecting less of them because of their disability (24%/19%), people believing that they were unable to make their own decisions (16%/15%), people ignoring or pretending not to see them (16%/15%), and people being awkward around them (15%10%) (*n* = 2,045/*n* = 895 disabled adults aged 16 and over in Great Britain). In the context of V.I., a UK survey of 769 blind and partially sighted people aged 13 and over found that 38% had experienced discrimination (RNIB, Guide Dogs, and TPT, 2022). More generally, people with V.I. may be more likely to experience discrimination than people with other disabilities (Flynn and Lord, 2015). For instance, after controlling for age and sex, adults with V.I. were found to be twice as likely as those with other types of impairments and over five times as likely as the UK general population to have experienced discrimination due to a disability (11% vs. 5 and 2% respectively) (McManus and Lord, 2012). In addition, 7% had been the victim of a hate crime. In England, older adults with poor eyesight were found to be more likely to have experienced discrimination than those with self-reported good eyesight (52.1% vs. 43.8%) (Jackson et al., 2019). This included being statistically significantly more likely within the past year to have been threatened or harassed (9.9% vs. 7.9%), treated with less respect or courtesy (36.3% vs. 31.0%), have received poorer service than other people in restaurants and stores (16.9% vs. 14.1%), and had people act as if they were not clever (23.0% vs. 18.1%) (Jackson et al., 2019). They were also significantly more likely to have ever received poorer service or treatment than other people from doctors or hospitals (20.4% vs. 16.3%). A 2015 telephone survey by the Royal National Institute of Blind People (RNIB) with over 1,200 blind and partially sighted adults aged 18 and over found that younger people and those registered as severely sight impaired or blind were more likely to have been treated unfairly because of their V.I., most commonly by strangers on the street (63%), retail staff (36%), bus drivers (23%), taxi drivers (14%) and health workers (14%) (Slade and Edwards, 2015). In addition, 10% of working-age and 3% of pension-age respondents had been the victim of a hate crime. Experiences of negative attitudes and prejudice may also be slightly but not significantly more prevalent among adults with V.I. from UK minority ethnic communities (63.7%) than white adults (58.5%) (Heinze and Castle, 2023).

The existing evidence suggests that people with V.I. continue to experience overt discrimination, ranging from poorer service in restaurants, stores and, critically, healthcare settings, to hostile and aggressive behaviours, as well as more covert behaviours arising from a lack of understanding of disabilities and the capabilities of people with disabilities. In addition to being treated differently, people with disabilities report a lack of social etiquette in interactions including being treated with less respect or courtesy, being stared at, strangers and friends addressing questions and comments to partners, friends or carers instead of them, being subjected to overly intrusive questioning including about their romantic relationships and fertility, being smothered with kindness (Grewal et al., 2002), or being ignored or treated as if they were invisible (Sanders, 2000).

It should be noted that the behaviours in these studies are self-reported rather than objectively measured. As such they reflect perceived discrimination. This may include instances where this reflects bad mood or disposition which results in, e.g., less respectful and courteous treatment of all people, rather than a dislike of people with V.I. Employment rates provide a more objective measure. Despite the introduction of laws that prohibit employment discrimination due to disability, people with V.I. remain underemployed relative to the UK general public (Slade et al., 2017). Research from the US suggests that employer attitudes relating to the productivity of employees with V.I. has the strongest association with employers’ intention to hire V.I. candidates (McDonnall and Lund, 2020). Other factors such as subjective norms (perceived support from the company, boss and employees for hiring a person with V.I.) and perceived behavioural control (perceived authority and ability to hire, and knowledge of where to find a qualified candidate with V.I.) were also found to play an important role in influencing hiring decisions (McDonnall and Lund, 2020). Similar research has not been conducted in the UK and it is therefore unclear to what extent these factors impact on hiring decisions relating to people with V.I. in the UK context.

Before reviewing the evidence relating to attitudes and perceptions towards people with V.I. which may drive these behaviours, it will be useful to understand how people think and feel about V.I. itself because negative connotations associated with ‘blindness’ may impact on perceptions of and attitudes towards those labelled as blind (Bolt, 2005).

## 4 Attitudes and perceptions of V.I.

Perceptions relate to how an object, person or group is seen or perceived. Public perceptions tend to be captured in large-scale surveys which aggregate individual opinions about a phenomenon into a public perception or opinion of the same. As such, public perceptions tend to reflect the normative, explicit beliefs of a group towards an object or group. Perceptions are often conceptualised as the cognitive component of attitudes (Dirth and Branscombe, 2018). The latter can be defined as a “relatively general and enduring evaluation,” positive, negative or neutral, of an object or people (Greenwald and Banaji, 1995; Briñol et al., 2019, p. 2), although the stability of attitudes is disputed (Wilson et al., 2000).

A mixed-methods study commissioned by the RNIB and Guide Dogs for the Blind Association asked participants to list 30 words which spontaneously came to mind when they thought of ‘blindness’ (Versiti, 2018). Blindness was associated with darkness (e.g., black, darkness), visible navigation aids (e.g., white canes, guide dogs), loss of functional ability (e.g., unable), dependence (e.g., help, reliance), negative affect (e.g., sadness, frustration, lonely, scared, anxious, angry), other senses (e.g., hearing, sound, smell, touch) as well as famous people with V.I. (e.g., Stevie Wonder, David Blunkett).

Although not the focus of their research, Biddyr et al. (2016) described how participants from different ethnic communities perceived sight loss as being devastating and resulting in dependence on others. Among the London Indian community, perceptions of deteriorating vision as being part of the ageing process, and sight loss as being a major disability only once it had led to significant dependence and loss of function resulted in delayed service use (Patel et al., 2006). This suggests that there may be slight differences in how V.I. is perceived among different groups. Furthermore, the extent to which V.I. is perceived to be a disability may impact on whether people are believed that they have a disability and whether they themselves identify as having a disability, and as a result access vital support and services. While 87% of British adults consider V.I. to be a disability (Robinson et al., 2007), only 50% of those with a sensory (hearing or visual) impairment consider themselves to disabled, although some of these identified as having their specific impairment or condition (Grewal et al., 2002)^1^. In other words, while people may self-identify as having a V.I. they may not identify as having a disability. The proportions considering ‘blindness’ to be a disability are relatively similar for respondents with (83%) and without disabilities (88%), with sensory impairments (84%), physical impairments (86%) and mental health conditions (89%), and with (88%) and without contact with someone with a disability (83%) (Robinson et al., 2007). Adults aged 65 and over were found to be least likely to consider V.I. to be a disability (81% compared to 90% of those aged 18–34) (Robinson et al., 2007). This may reflect the prevalence of V.I. in the older age group, who may not feel disabled by their condition, consider it part of the ageing process, or be reluctant to self-identify as having a disability.

Sight is the most valued sense among the UK public followed by hearing (Enoch et al., 2019). In a discreet choice experiment, UK adults were prepared to sacrifice 5.4 years of their life to avoid sight loss but only 1.4 years to avoid hearing loss, although the authors contend that this may reflect the cultural dominance of sight in English-speaking societies (Hutmacher, 2019; Enoch et al., 2020). A survey of 260 UK teenagers aged 16–18 years showed that they were significantly more fearful of developing V.I. than other conditions such as lung cancer, heart disease and deafness (Moradi et al., 2007), however, a survey using the same questions with adults aged 18 and over found that they were significantly less fearful of developing V.I. than lung cancer, heart disease and stroke (Bidwell et al., 2005). Shakespeare (1994) cautions that much of the fear may relate to an unrealistic perception of what life with a disability is like, nurtured by cultural representations of disability which emphasise dependence and inability. Indeed, UK adults have raised concerns about the absence of people with disabilities in the media, and the focus on their disabilities rather than their normal lives where they appeared in TV programmes or films (Grewal et al., 2002). Media portrayals of people with disabilities have tended to fall into either charity or celebrity images. While charity images elicit sympathy by portraying people with disabilities as needing support from others and being unable to do things for themselves, celebrity images portray people with disabilities as courageous and brave, having to overcome adversity to live normal lives (Grewal et al., 2002).

People with V.I. have identified a lack of awareness of V.I. and its impact as a main barrier to their participation in everyday life (Fisher et al., 2018) and a reason for social isolation and discrimination (Slade and Edwards, 2015). Indeed knowledge of V.I., including eye conditions which may cause V.I., and risk factors for these conditions is relatively poor (Shickle et al., 2014; Shickle and Griffin, 2014). As described earlier, V.I. tends to be equated to blindness without light perception. This may impact on those living with partial V.I. (Dale, 2010; Nyman et al., 2012), who have enough residual vision to navigate their environment without white canes or guide dogs. Awareness of the risk of V.I. from smoking has been found to be as low as 9.5% among UK adults aged 18 and over (Bidwell et al., 2005) and 5% among teenagers aged 16–18 (Moradi et al., 2007). Qualitative research with 48 Afro-Caribbean participants in the UK found relatively high awareness but limited understanding of primary open-angle glaucoma (Cross et al., 2005), an eye condition which may result in V.I. and is believed to be more prevalent among people of Afro-Caribbean heritage across all age groups (Wormald et al., 1994; Rudnicka et al., 2006). Descriptions of glaucoma were generally vague (“*something to do with skin over the eyes*,” p.84) but more detailed (“*build-up of pressure due to lack of drainage*,” p.84) if people had heard about the condition from a primary care provider. There was also a lack of awareness of risk factors: Cross et al.’s participants perceived glaucoma to be a “*blinding condition of elderly people*” (p.85) resulting in a sense of invulnerability among younger participants (Cross et al., 2005). Beyond awareness of eye conditions, a lack of awareness of how to behave around working dogs can result in restaurants and shops barring guide dogs from entering, or members of the public distracting guide dogs by wanting to pet or feed them (Sanders, 2000).

Although a formal assessment of eye knowledge among the UK general public has not been conducted, the available evidence seems to suggest that overall, awareness and knowledge of V.I. and different eye conditions, even among high-risk groups, is limited. The lack of awareness may be due to ineffective or missing public health information around V.I. and/or a lack of exposure to people with disabilities in the public domain, media and in everyday life. For example, the proportion of people who knew someone with disabilities cited in previous research has ranged from around 9 in 10 (Grewal et al., 2002) to 48% (Cant and Bennett, 2022). A lack of knowledge may result in V.I. being associated with fear, a loss of independence and social exclusion. These perceptions and attitudes may in turn impact on the way people with V.I. are perceived.

## 5 Attitudes and perceptions – how the public feel and think about people living with V.I.

A 2022 UK survey of 4,015 adults with different disabilities found that around 2 in 5 (42%) had experienced negative attitudes from the general public and other passengers on public transport (39%) (Moss and Frounks, 2022). In addition, between a quarter and half of respondents had experienced negative attitudes from a range of support staff including benefit assessors (52%), job centre staff (46%), social workers (36%), clerical health staff (33%), carers/personal assistants (29%), GP medical staff (29%), specialist healthcare staff (25%) and police/emergency services (25%) (Moss and Frounks, 2022). Furthermore, at least 1 in 5 had experienced negative attitudes from call centre (29%), retail (28%), airport (26%), leisure (26%), hospitality (25%), bus (25%), rail/train staff (25%) as well as taxi (24%) and delivery drivers (21%) (Moss and Frounks, 2022). In the context of work, around 2 in 5 had experienced negative attitudes from management (42%), colleagues (41%), and recruitment agency staff (40%) (Moss and Frounks, 2022). In the context of education, around a third had experienced negative attitudes from other students (37%), careers advisors (36%) and teachers, lecturers, or other training staff (35%) (Moss and Frounks, 2022). Of note is the finding that 29% had experienced negative attitudes from family, 27% from partners and romantic relationships and 25% from friends, all of whom constitute a person’s informal support network (Moss and Frounks, 2022).

Attitudes toward people with disabilities may be shaped by underlying models of disability. The medical model of disability construes impairments as medical conditions which need to be treated and cured (Grewal et al., 2002). This association with disease and illness can be problematic due to evolved disease avoidance mechanisms which may trigger emotions such as disgust and anxiety, and result in negative attitudes and avoidance (Park et al., 2003). In contrast, the social model of disability distinguishes between impairment and disability and views disability as arising from social (i.e., environmental and attitudinal) barriers (Grewal et al., 2002; Bolt, 2005; Shakespeare, 2006). A third model, the charity model of disability, construes disability as a personal tragedy which is to be overcome, eliciting helping behaviours and pity, on the one hand, but also admiration for the perceived courage of those living with disability (Grewal et al., 2002) and fear (Enoch et al., 2020), on the other hand. Grewal et al. (2002) found that British people’s attitudes and perceptions of people with disabilities were also shaped by the media, direct or indirect personal experiences of disabilities, and family while growing up. Behavioural responses to individuals with disabilities may therefore consist of evolved (Park et al., 2003) and learned elements. The learned aspect of people’s attitudes and reactions is manifested by a respondent in Grewal et al.’s (2002) work on attitudes and perceptions towards disability:

M1 ‘… and then your parents when you see somebody in a wheelchair, walking along as a kid with your parents saying don’t stare, don’t stare, it’s not good to stare, and so it’s this disconnection that makes things [difficult] generally people, when you become young adults or adults, it’s an awkwardness, it’s not necessarily they’re not normal but it’s an awkwardness of I don’t really know how to approach this person so therefore I will steer clear of the situation and I think it’s a personal awkwardness rather than I think that this person is abnormal. And it all stems from a lack of interaction.’ (p.47)

In the context of V.I., a 2015 telephone survey by the RNIB found that over a third (35%) of their 1,223 blind or partially sighted respondents had experienced negative attitudes at least sometimes in the last 12 months because of their V.I. (Slade and Edwards, 2015). Younger people and those registered as severely sight impaired or blind were more likely to report experiencing negative attitudes by the general public. This is slightly lower than the proportion (46%) who agreed that the UK general public is often prejudiced against people with V.I. reported elsewhere (RNIB, Guide Dogs, and TPT, 2022).

**Figure 1:**
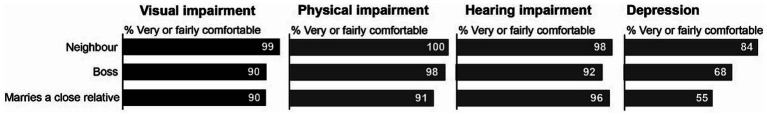
Shows the proportion of BSA 2005 respondents who would feel very or fairly comfortable with interacting with people with different types of disabilities in different scenarios (adapted from Robinson et al., 2007).

However, research on public attitudes towards and perceptions of people with V.I. from the perspective of the UK public is limited. Mixed-methods research with 1,000 UK adults found that people who are blind are perceived to be somewhat more conscientious (more careful, organised and self-disciplined) and less neurotic (more calm, emotionally stable and secure) than those with partial sight and those without V.I. (Versiti, 2018). In addition, at least two thirds of respondents implicitly associated people with blindness with being resilient, resourceful, genuine, determined, courageous, skilled, calm, brave, but also with being quiet and vulnerable. People with partial sight were associated with being capable, independent, strong, resilient, determined, brave, resourceful, positive, skilled, courageous, and genuine, but also with being vulnerable. Perceptions were markedly more negative for those living with depression and autism. Similarly, Grewal et al. (2002) asked 1,099 UK survey respondents with and 965 without disabilities to describe how they would feel if they came across a person with a guide dog in a restaurant and the guide dogs was getting restless and starting to sniff around. Most participants reported that they would feel fine/not bothered (57%), understanding/genuinely concerned (32%) and sorry for the person (30%). Only small proportions would feel worried about adverse reactions/offending the person (3%), surprised/shocked (3%), annoyed/irritated (4%), and nervous/anxious (4%). Around 66% of the non-disabled respondents stated this situation would not bother them, 30% would feel understanding/concerned and 18% would feel sorry, while 1%, respectively, would feel annoyed, threatened, suspicious and anxious/nervous. The British Social Attitudes survey (BSA) is a large-scale annual general population survey which explores social attitudes relating to a wide range of varying topics. The 2005 edition explored disability attitudes among a nationally representative sample of 3,193 adults^2^ aged 18 and over living in Great Britain (GB) (Robinson et al., 2007). Prejudice against people with V.I. was perceived to be lower than for any other condition: 1 in ten (10%) respondents thought there was ‘a lot’ of prejudice against people with V.I. (compared to 13% for hearing impairment, 20% for physical impairment, 25% for disability in general and 46% for schizophrenia) and 1 in 5 (21%) thought there was ‘none’ (compared to 14% for hearing impairment, 10% for physical impairment, 8% for disability in general and 4% for schizophrenia). Moreover, respondents generally felt comfortable about interacting with people with V.I.: at least nine in ten felt very or fairly comfortable having a boss with V.I. (90%), having their close relative marry someone with a V.I. (90%), and having someone with V.I. as a neighbour (99%). Respondents with a disability and those who knew someone with a disability were more comfortable, although this was not statistically significant. The proportions are relatively similar for people with physical and hearing impairments, but lower for people with mental health conditions such as depression (Figure 1). While 79% felt very comfortable about having a neighbour with V.I. and 61% about having a boss with V.I., only 51% felt very comfortable about a person with V.I. marrying their close relative. This suggests that people would feel comfortable interacting with a person with V.I. within their community and at work but less so in the family. There is evidence that adults with disabilities such as V.I. may be perceived as being asexual (Milligan and Neufeldt, 2001; Kim, 2011) and may not be considered acceptable as romantic partners. For instance, sighted college students reported that their peers would be less comfortable and less likely to date a peer with V.I. than a sighted peer, possibly due to fear about what friends might think (Fichten et al., 1991). In addition, research from Israel found that women with V.I. from traditional Muslim families experienced stigma relating to their acceptability as a romantic partner from potential partners, the partner’s family and their own families (Shpigelman and Vorobioff, 2019).

In contrast, timed sorting exercises found that people with blindness were perceived to be slightly more suited to family roles including lover (although less to being a spouse or sibling) than most work roles with the exception of musician (Versiti, 2018). The exercises asked 1,000 UK adults to assess how suited people with different disabilities were to different social roles and contexts within a short time limit to minimise social desirability bias. Overall, ‘blindness’ was perceived to be least compatible with most social roles and contexts compared to other disabilities including partial sight (Versiti, 2018). People with blindness were perceived to be more suited to places of worship, pubs and discos than any of the other contexts. People with partial sight were perceived to be compatible with most family and work roles but less so with different environments, particularly art galleries/museums, libraries, mountain trails and, interestingly, taxis. Long-term health conditions such as diabetes and asthma were generally perceived to be more compatible with all social roles and contexts.

The 2021 edition of BSA assessed work-related attitudes towards people with V.I. and other disabilities, including a revised question about the acceptability of a boss with disabilities. Just over two thirds of BSA 2021 respondents were comfortable having a colleague (69%) and boss with V.I. (67%) (Cant and Bennett, 2022). The latter is substantially lower than the proportion recorded in 2005 (90%) which may reflect changes in the question wording or changes in attitudes. Attitudes towards a boss with V.I. were somewhat more positive among younger adults and those educated to degree level, but there were no differences for sex nor employment status. As in 2005, attitudes were more positive towards workers who have V.I. and mobility impairments, than towards those with mental health conditions such as depression or schizophrenia. Despite the general acceptance of colleagues with V.I., only 41% thought that people with V.I. were perceived by their fellow workers as doing just as good a job most of the time (compared to 40% for people with mobility impairment, 20% for those with depression and 15 for those with schizophrenia).

Babik and Gardner (2021) reviewed evidence relating to factors that impact on attitudes towards people with disabilities and traced the development of disability perceptions in children: children start to display negative attitudes towards an out-group by the age of 7 as they become socialised and aware of social groups, although prejudice may develop earlier among children who grow up in homogenous environments. Explicit negativity may reduce with age as children became more reliant on personal experiences of contact with outgroup members and aware of social norms which proscribe prejudice and discrimination. Although, the latter is likely to reduce explicit but not implicit negative attitudes (Babik and Gardner, 2021). The authors suggest that education about disabilities as well as fairness and equality may reduce generalisations (such as conflating physical impairments with cognitive deficit) and negative attitudes in children. Other factors which are proposed to impact on disability perceptions included cultural norms and beliefs, parental attitudes and practices, the child’s personality, empathy and sympathy, theory of mind (i.e., the child’s ability to understand that other people may have thoughts, beliefs, knowledge and intentions that are different to their own), self-esteem, gender, attachment styles, and exposure (Babik and Gardner, 2021).

Nikolaraizi and De Reybekiel (2001) explored attitudes towards children with hearing, visual and physical impairments in a sample of children aged 10–12 years attending primary schools in the UK (*n =* 234) and Greece (*n =* 229). Overall, attitudes were positive among all children in this study, but this did not necessarily translate into a willingness to form closer relationships with children with impairments. Greek children (relative to British children) and girls (relative to boys) held significantly more positive attitudes overall towards all three groups. Among children in the UK, attitudes towards children with V.I. were slightly less positive than towards the other two groups. British children were significantly more likely than Greek children to care if other children made fun of a child with V.I., but they were less likely to ask a child with V.I. to sit beside them, chat with them at breaktime, make them their best friend and think that they could be taught in the same classroom as them. Although attitudes were generally positive on all questions, British children were also significantly more likely than Greek children to be scared of a child with V.I. and to think that blind children prefer to be with other blind children. The authors propose that the children express socio-emotional concern but less willingness to befriend children with V.I. because they hold parental attitudes which result in protective behaviours, are afraid of the unknown and feel insecure, and/or do not want to admit negative attitudes towards children with V.I.

## 6 Discussion

There are several conclusions to draw from the evidence described above. First, more research is required to establish the exact content of attitudes and perceptions, how these shape behaviours, and how behaviour in turn shapes attitudes. Second, there is a perception gap, i.e., a gap between the perceived attitudes and discrimination reported by people with V.I. and the attitudes expressed by members of the UK public. For instance, while there is some evidence that people with V.I. were more likely to have experienced discrimination than those with other impairments (McManus and Lord, 2012; Flynn and Lord, 2015), the British public believe that prejudice against people with V.I. is less prevalent than for people with other conditions (Robinson et al., 2007). Moreover, the limited research available for the UK shows that attitudes were largely positive, with large majorities reporting that they would feel very or fairly comfortable interacting with people with V.I. in different social contexts. This contrasts the experiences reported by people with V.I. which range from overt to more covert negative behaviour.

### 6.1 The perception gap

There are several possible reasons for this. A number of models have been proposed to explain attitudes, emotions and behaviours towards minority groups. Fiske et al. (2007), for instance, proposed that humans have evolved to evaluate others on two dimensions: warmth (including fairness, friendliness, helpfulness, righteousness, honesty, sincerity, tolerance, understanding, trust, morality, and generosity) and competence (intelligence, cleverness, skill, creativity, efficiency, efficacy, foresightedness, ingenuity and knowledge). First, the other’s intention towards one’s own group (i.e., perceived warmth) is assessed. This results in a positive or negative evaluation (or liking) of the other. Then the other’s skill and ability to enact those intentions (i.e., perceived competence) is assessed, impacting on how negatively or positively the other is evaluated. Perceptions on these two dimensions are proposed to constitute the content of stereotypes and elicit specific emotions and behaviours. Stereotypes constitute beliefs about the traits and characteristics, positive and/or negative, which are thought to be typical of a social group and therefore generalised to all members of this group (Greenwald and Banaji, 1995). Greenwald and Banaji (1995) give the example of stereotypes relating to cheerleaders which contain both positive (physical attractiveness) and negative elements (lack of intelligence). At the implicit level, stereotypes are thought to be shared even by members of the stereotyped group, such that women and men both hold implicit gender stereotypes (Jost and Banaji, 1994). People with disability tend to be perceived as being high in warmth and low in competence (Fiske et al., 2007). The ‘behaviours from intergroup affect and stereotypes’ (BIAS) map predicts that groups who are perceived as high in warmth and low in competence elicit pity and sympathy (emotions) which may result in active helping but also exclusion or neglect (behaviours) (Cuddy et al., 2007; Fisher et al., 2018). While people with disabilities are indeed perceived to be warmer but less competent than those without disabilities on explicit measures, implicit measures indicate that people with disabilities are perceived as less competent as well as less warm (Rohmer and Louvet, 2012). Follow-up research showed that people with disabilities were consistently associated with less warmth regardless of context, but as less competent only in the context of work (Rohmer and Louvet, 2018).

The difference in reported attitudes and observed behaviours may therefore reflect a “dissociation” between explicit and implicit attitudes (Greenwald and Banaji, 1995, p.8) towards those with V.I. British adults were relatively positive when asked how comfortable they would feel about interacting with V.I. in various social roles, but attitudes were less positive when asked to assess the suitability of people with V.I. for various social roles under time pressure. In other words, people with V.I. are assessed as competent when asked explicitly but not implicitly. Explicit attitudes are thought to require motivation and cognitive capacity to retrieve from memory and, as such, are receptive to change, while implicit attitudes are thought to be activated automatically, operate outside the control of people, and be relatively resistant to change (Wilson et al., 2000). Data on explicit attitudes is collected using a variety of methods including opinion surveys, interviews, scales (e.g., rating or social distance scales) or Q methodology (sorting exercise based on favourability, intensity of agreement, descriptiveness). These can be subject to various biases, social norms as well as laws (Antonak and Livneh, 2000). Mixed attitudes, which include positive and negative evaluations, expressed at the explicit level may therefore reflect societal norms and social desirability bias (Rohmer and Louvet, 2012). In contrast, people are not always aware of their implicit attitudes. Various techniques have been used to assess implicit attitudes towards people with disabilities including behavioural observations, projective techniques (e.g., word association, story or sentence completion tasks), disguised procedures or techniques (e.g., using photos or vignettes, obscuring/hiding the purpose of the study), physiological methods (e.g., skin conductivity/resistance, pupil dilation, heart rate, pulse and blood pressure) (Antonak and Livneh, 2000), timed exercises such as the one described above (Versiti, 2018) which use time restraints to elicit automatic rather than controlled responses, and the implicit association test (IAT) (Wilson and Scior, 2014). A literature review found moderate to strong implicit negative attitudes towards people with disabilities, which were either not or only weakly correlated with explicit attitudes (Wilson and Scior, 2014). This indicates that people may hold more than one attitude towards the same object, which may coincide, conflict or coexist (Wilson et al., 2000). According to dual-processing models of attitudes, behaviours and other responses depend on the extent to which people are aware of their implicit attitudes, and the extent to which they are motivated and have the capacity and time to override these (Wilson et al., 2000). Dovidio et al. (2011) reviewed existing evidence which showed that explicit attitudes impact on behaviours which can be monitored and controlled such as guilt and attractiveness ratings, while implicit attitudes may impact on more automatic, nonverbal behaviours. For instance, Rojahn et al. (2008) found no difference in the romantic attractiveness ratings of people with and without physical disabilities, despite a negative implicit attitude towards those with disabilities. Elsewhere, Scottish teachers’ explicit but not implicit attitudes towards children with intellectual disabilities predicted self-reported inclusive teaching behaviours, although this was partially mediated by self-efficacy (Wilson et al., 2022). Explicit and implicit attitudes were not correlated, and implicit attitudes were more positive in teachers who had received special education training (Wilson et al., 2022). Similarly, explicit but not implicit attitudes were associated with emotional reactions and social distance to people with intellectual disabilities (Wilson and Scior, 2015). Explicit and implicit attitudes were again not correlated, with implicit attitudes being somewhat negative but explicit attitudes being positive. While there were no differences in implicit attitudes relating to demographics or contact with people with intellectual disabilities, women and those with higher educational attainment had more positive explicit attitudes. The behaviours in these studies relied on self-report and were therefore subject to the same reporting biases as explicit attitudes.

Parallels can be drawn with research into racism. Dovidio and Gaertner (1986, p. 62) propose that aversive racism constitutes a modern form of racism in which egalitarian beliefs and attitudes conflict with “unacknowledged” negative beliefs and attitudes towards Black individuals, replacing more overt, hostile racism. As a result, explicitly expressed attitudes towards Black people may be positive but Black people may continue to experience subtly discriminatory behaviours. An experimental study found that White students’ explicit attitudes towards Black people predicted their self-perceived and verbal friendliness in interactions with Black confederates, while implicit attitudes were associated with non-verbal friendliness, as well as friendliness ratings by confederates and observers (Dovidio et al., 2002). Furthermore, activation of stereotypes may trigger stereotype-consistent behaviours which can be transferred to an interaction partner, thus confirming stereotypes in interactions. For instance, Chen and Bargh (1997) showed how activation of stereotypes relating to African Americans in one interaction partner resulted in more hostile unconscious behaviour in both interaction partners. In the context of V.I., it is therefore possible that discriminatory behaviour experienced by people with V.I. is driven by non-verbal behaviours elicited by negative implicit attitudes which operate outside of the conscious awareness of the public. Moreover, stereotypes which view people with V.I. as less capable may trigger behaviours that would be experienced as infantilising or patronising by adults with V.I. resulting in anger and frustration and, ultimately, negative interaction experiences for both individuals. Stereotypical beliefs about a group may be acquired at an early age and can be activated automatically to make social judgements about a group or group member (Devine, 1989, 1995). Non-prejudiced behaviour and evaluations then require active suppression or control of stereotypes. Attitudes and behaviours may also depend on the extent to which they deviate from stereotypes of what it is to be a typical member of a majority group. For instance, blindness was associated with being a victim, helplessness and social isolation in a series of interviews and focus groups with 48 adults from Afro-Caribbean communities (Cross et al., 2005). This conflicted with cultural stereotypes which described Afro-Caribbean people and particularly women as being proud, stoic and independent and may result result in a reluctance to talk about V.I. (e.g., to share family histories of V.I.), seek a timely diagnosis and/or treatment, and use mobility aids such as guide dogs or canes.

Overall, this suggests that the public may not always be aware of their attitudes and behaviours when interacting with people with V.I. Second, it highlights that the drivers of behaviour are complex. Although attitudes play a role in behaviour, this relationship is not necessarily linear (Bechler et al., 2021). Thus, more negative attitudes do not necessarily result in more negative behaviour.

Alternatively, perceptions of negative attitudes may relate to a misunderstanding between interaction partners. Ajzen (1991) suggests that attitudes (as well as personality traits) are a poor predictor of behaviours in specific contexts, instead attitudes may be a better predictor of aggregate behaviours across different situations and contexts, as this would remove situational influences. While members of the UK public may therefore hold positive attitudes towards people with V.I., these are not always manifested in individual social encounters. In other words, negative attitudes and behaviours reported by people with V.I. may reflect negative experiences in individual social interactions, rather than an overall negative predisposition among the public. Moreover, lay understandings of discriminatory behaviour may diverge from existing legal definitions of what constitute discrimination and what does not, making this distinction less clear in everyday interactions. This is illustrated by the example of a man opening the door for a woman, a gesture considered sexist by some groups and polite by others (Demoulin et al., 2013). Thus, misunderstandings may arise from different perspectives of what is discrimination and what motivates a behaviour (such as opening a door).

Furthermore, sighted people may experience conscious or unconscious anxiety or awkwardness in anticipation of, and throughout, an interaction due to a lack of understanding of V.I., a conflict between implicit and explicit attitudes, and/or a desire to avoid insulting the person, which may result in avoidance and non-verbal cues of anxiety. There is evidence of the negative impact of intergroup anxiety on attitudes towards people with disabilities (Byrd and Zhang, 2020). In the context of race, people who expected interactions with Black people to be negative reported higher intergroup anxiety (Plant, 2004). This was in turn associated with a greater desire to avoid interactions with Black people in those who were not personally motivated to interact without prejudice (Plant, 2004). Pearson et al. (2008) suggest that due to the similarity between non-verbal cues of anxiety and negative attitudes, anxiety (for instance in sighted people) may be interpreted as aversion. Non-verbal cues of anxiety may also be transferred to a person with disabilities though emotion contagion. Emotion contagion is the synchronisation with the emotional state of an interaction partner (Herrando and Constantinides, 2021). It can be triggered physiologically or neurologically, in direct and indirect social interactions, e.g., by a facial expression and/or a person’s behaviour. Social interactions with an anxious sighted person may be experienced as uncomfortable/unpleasant by both interaction partners. Moreover, interactions marked by anxiety and a mismatch between verbal and non-verbal behaviours and cues may result in mistrust and heightened awareness of further negative cues resulting in the stereotype confirmation described earlier (Chen and Bargh, 1997). Dovidio et al. (2011) speculate that mismatches in verbal and non-verbal behaviours may occur due to a focus on controlling verbal behaviours (e.g., expressions of sympathy and support), limiting the cognitive resources available to control non-verbal behaviours such as avoiding eye contact, closed posture and distance which convey anxiety. Judgements of others’ behaviour are impacted by a number of biases including attribution bias which relates to a tendency to attribute outcomes to dispositional rather than situational factors (Ross, 1977). In other words, there may be “a tendency to perceive even neutral behaviours displayed by nondisabled individuals as discriminatory actions against their stigmatised status” (Hebl and Kleck, 2000, p. 424). Although a person’s own perceptions and attitudes towards people with V.I. prior to acquiring V.I. may also impact on how they perceive social encounters. However, research to date has not explored to what extent negative experiences reflect actual or perceived discrimination and prejudice.

Implicit attitudes and the contagion of anxiety and discomfort in interactions may together, or independently, impact on interaction experiences between people with and without V.I. and, as such, impact on perceived discrimination and attitudes. Future research will need to explore their respective roles and the scope for interventions to change interaction experiences. The following provides some additional considerations for future research.

### 6.2 Other considerations

Social representations theory posits that knowledge construction and reconstruction involve a dynamic process of meaning-making among the members of a group. As such, knowledge is not stable and static but rather dynamic and contested (Moscovici, 2000). New information or experiences can impact on the meaning associated with an object or group. In other words, learning about V.I. and positive experiences with people with V.I. can impact on how they are perceived. Social norms and legislation have improved explicit attitudes and behaviours. However, due to their receptiveness to change, attitude change initiatives tend to impact on explicit rather than implicit attitudes. For instance, a curriculum-based educational intervention improved explicit but not implicit attitudes towards children with disabilities among primary students (Wüthrich et al., 2023). As seen earlier this may in turn impact on explicit behaviours but not implicit non-verbal behaviours. Greenwald and Banaji (1995) suggest that attention may weaken the impact of implicit attitudes. This would correspond with the notion that behaviour is contingent on people’s awareness of their implicit attitudes and their motivation and capacity to override them (Wilson et al., 2000). Consciousness-raising may therefore be one strategy to reduce discrimination, while affirmative action may not only provide compensation for explicit and implicit prejudice, but also be more easily applied in a real-world context such as employment than a third strategy, blinding, which relies on hiding attributes which may elicit implicit reactions such as the gender or ethnicity of a job applicant (Greenwald and Banaji, 1995).

There is further evidence that intergroup contact may be effective in changing negative attitudes towards a stigmatised group. The BSA 2021 showed that attitudes towards people with V.I. were more positive among those who had contact with someone with an impairment (Cant and Bennett, 2022). A systematic review found that the frequency and quality of contact with people with disabilities as well as knowledge of disabilities may be associated with more positive attitudes towards those with disability (Wang et al., 2021). Interventions which involve interactions with peers with disabilities have been found to be more effective in changing attitudes than educational interventions in adolescents (Mpofu, 2003; Krahé and Altwasser, 2006). Noteworthy are findings from Nikolaraizi and De Reybekiel’s (2001) work with Greek and British school children: attitudes towards children with V.I. were more positive among British children attending schools with than those attending schools without special needs units, but the reverse was true in Greece. Moreover, there is mixed evidence relating to which attitudes are impacted by contact. Hein et al. (2011) found that the amount (but not quality) of contact was associated with the affective, cognitive, and behavioural components of explicit, but not implicit attitudes. In contrast, Wilson and Scior (2015) found that implicit attitudes became more positive as contact increased (up to 1–2 times per week). No differences were found between those who had daily contact and those who had only infrequent contact, suggesting there may be a plateau effect of contact. Byrd and Zhang (2020) similarly found an indirect effect of contact frequency (via social support and intergroup anxiety sequentially) and contact quality (via intergroup anxiety) on attitudes and stereotype endorsement, indicating that repeated positive intergroup contact may provide opportunities to challenge stereotypes and lower anxiety which in turn impact on attitudes.

There may be other aspects which impact on contact quality. For instance, distraction may inhibit stereotype activation but increase stereotype application, suggesting that distraction may reduce the cognitive resources available to retrieve stereotypical beliefs from memory (Gilbert and Hixon, 1991). In the context of race, Plant (2004) found that people who were internally motivated to respond without prejudice had more positive outcome expectations in relation to social interactions with Black people, less intergroup anxiety, and a lower desire to avoid interactions than those who were less motivated. In contrast, external motivations (e.g., to avoid social disapproval) were marginally associated with higher anxiety (*p* = 0.09). In other words, interaction expectations and outcomes may be more negative in people who do not want to be seen as being prejudiced (i.e., external motivation) than those who do not want to be prejudiced (i.e., internal motivation).

Similarly, the interaction context may be important: ambivalence-amplification theory (Katz, 1981; Katz et al., 1986) proposes that ambivalence arising from associating both positive (sympathy) and negative (aversion) elements with stigmatised groups results in amplified responses to members of these groups. In other words, people will respond more positively towards a member of a stigmatised group in a positive context but more negatively in a negative context than towards a member of a non-stigmatised group. For instance, Bromgard and Stephan (2006) found that their male participants sat further away from a conversation partner who identifies as gay than they did from non-stigmatised conversation partners when they anticipated a potentially threatening conversation, but they sat closer when anticipating a non-threatening conversation. Since there were no differences in explicit attitudes, the authors propose that amplified responses may be most visible in non-conscious behaviours. The context in which interactions between people with and without V.I. take place may therefore impact on behaviours.

Attitude towards a person with V.I. may also depend on how they are categorised. For example, implicit attitudes towards Michael Jordan were found to be positive when the category ‘athlete’ was primed, but they were negative when priming the category African American (Mitchell et al., 2003). Indeed, when the occupation was primed, participants preferred a list of popular Black athletes to a list of disliked white politicians, but when race was primed, participants preferred the disliked white politicians to the popular Black athletes (Mitchell et al., 2003). Although the sample size was small (*n* = 10 White female university students), implicit attitudes were found to shift even when the contrasting group was not explicitly primed but instead one feature (e.g., race or gender) was made salient in the contrasting group. For instance, evaluations of Black women became as positive as they tend to be for women when the task included male distractors, but they became as negative as they tend to be for Black people when the task included White distractors. This shows how different categorisations can change attitudes towards the same person and warrants further exploration to see if negative attitudes towards people with V.I., where they exist, can be changed in interactions by priming a group associated with more positive attitudes.

This article has focused on social psychological theories which may serve to explain the experiences of people with V.I. However, work in other areas may shed further light on what drives behaviour. For instance, two theories from the field of behavioural science provide some insights into the factors that might motivate behaviour. In the COM-B model (Michie et al., 2011), behaviour is proposed to arise from the interaction of capability, opportunity, and motivation. The model has been used to explain physical activity and eating behaviour in young adults (Willmott et al., 2021), and may serve to explain some of the findings described in this article. For example, a lack of awareness of how to behave around working dogs (i.e., capability) may result in members of the public wanting to pet or feed a guide dog whilst it is working (Sanders, 2000). Similarly, when asked how they would feel about sitting next to a person who was blind at a dinner party, UK adults reported feelings of anxiety, confusion and awkwardness resulting from uncertainty about what to say and how to act (i.e., capability) (Versiti, 2018). Meanwhile, the theory of planned behaviour (TPB) (Ajzen, 1988, 1991) is based on the premise that behaviours in a specific situation are impacted by a person’s perceived ability (perceived behavioural control) and motivation (intention) to perform them. Ability relates to the extent to which people have perceived and actual control, i.e., the opportunity and resources including skills, money, time, cooperation of others, to behave in a certain way. Intentions express people’s motivations to behave a certain way and are determined by attitudes towards the behaviour, subjective norms and perceived behavioural control. In other words, behaviour in a specific context is shaped by how a person is disposed towards a behaviour, how much control they feel they have to perform the behaviour and how much social support there is for the behaviour. This was seen in the hiring decisions of US employers (McDonnall and Lund, 2020), which were impacted by subjective norms and perceived behavioural control as well as attitudes.

Seen through the lens of the TPB and COM-B models, explicit positive attitude (i.e., feeling comfortable) may result in an intention or motivation to support colleagues or a boss with V.I., however, this may not necessarily translate into behaviour if they feel unable to do so (i.e., perceived behavioural control), for instance, if they do not have the knowledge or skills (i.e., capability) to offer assistance or make reasonable adjustments, if there are competing subjective norms (i.e., helping people with disabilities and not offending people with disabilities), or if they do not have the opportunity in the external environment to perform the behaviour (e.g., because they are under time pressure at work, or spend little time with their colleague with V.I.). Research on physical activity (Rhodes and de Bruijn, 2013) and ethical consumption (Hassan et al., 2016) suggests that there can be a gap between intentions and behaviours. However, the behaviours reported by people with V.I. can be more covert than more observable behaviours, such as physical activity or recycling, and may therefore be more difficult to assess. Indeed, there are differences in the extent to which models such as the TPB can explain different types of behaviours (McEachan et al., 2011). Future research will need to identify the factors which impact on the behaviours reported by people with V.I.

A final but important consideration is the role of intersectionality for those who belong to more than one stigmatised group. In relation to employment, for instance, V.I. has been associated with poorer employment outcomes, including a greater risk of being unemployed or unable to work, in a lower-status job, and in the lowest income bracket (Cumberland and Rahi, 2016). Although there is considerable variation between different ethnic communities and despite a steady increase in employment rates among adults from minority ethnic communities since 2012, 67% of adults from minority ethnic communities in Britain were in employment in 2021 compared to 76% of white adults (ONS, 2022). Moreover, V.I. has been associated with poorer mental health outcomes (Kempen et al., 2012; Zhang et al., 2013; van der Aa et al., 2015; Frank et al., 2019), but there is also evidence of mental health inequalities among adults from minority ethnic communities in the UK in terms of diagnoses of mental health conditions and experiences of mental health support services (Grey et al., 2013; Hussain et al., 2022). Research with adults with disabilities who are LGBTQI+ found that they may feel uncomfortable to disclose their status to authorities and personal carers, due to concerns that their status may impact on the care provided to them if an assessor or personal carer has strict religious beliefs (Abbott et al., 2017). Over half of participants in this study never or only sometimes disclosed their LGBTQI+ status to their personal carer, under a third felt comfortable discussing their needs as a LGBTQI+ person and around one in five did not receive help to take part in LGBTQIA+ activities. While 40% had not disclosed their LGBTQI+ status to their personal assistant and 40% did not feel comfortable receiving help with this from them, 20% reported that their personal assistant had refused to help them with this. Moreover, they described experiences of abuse, discrimination and being outed to family members without their consent, at times due to strict religious beliefs of personal carers and local authority staff (Abbott et al., 2017). Although not exhaustive, these examples provide an insight into the unique difficulties experienced by adults who have a disability and belong to another stigmatised group.

Although community-specific attitudes are an underexplored area, there is evidence of the presence of multiple social representations of people with V.I., resulting in a state of cognitive polyphasia (Jovchelovitch, 2002; Provencher, 2011). Cognitive polyphasia refers to the co-existence of, at times diverging and incompatible, knowledge systems including attitudes, beliefs and norms within the same group or even individual (Renedo and Jovchelovitch, 2007). Cognitive polyphasia emerges from the encounter of different knowledge systems (Jovchelovitch, 2019), e.g., through migration. For instance, Higginbottom et al. (2014) found highly stigmatised perceptions of people with V.I. as not existing and not being able to do anything for themselves among Somalian refugees in the UK. These resulted in learned helplessness, and a reluctance to self-identify as visually impaired and access vision rehabilitation and support services available in the UK (Higginbottom et al., 2014). Encounters between conflicting knowledge may play out in different ways: conflicting knowledge may be rejected and displaced, anchored in an existing representation to form a hybrid representation, or co-exist independently and used to solve different problems. Denise Jodelet (1991) studied social representations of madness in a French village which took in adults with mental health difficulties as paying lodgers. Representations of lodgers as innocent, child- or animal-like coexisted with representations of madness as disease and otherness, resulting in practices which strictly separated the food, dishes and laundry of the lodgers from those of the villagers. The Chinese community in Britain uses a hybrid representation to address different health problems: whereas traditional Chinese methods are used to treat minor health issues that involve little or no pain and to treat the cause of an illness, biomedical solutions are sought to treat more serious health conditions and those involving pain (Gervais and Jovchelovitch, 1998). Similarly, Wagner et al. (1999, 2000), found that educated, middle-class residents in India drew on scientific representations of madness and psychiatry to enable communication in the context of the research interview, but on traditional representations of madness and healing methods favoured by their family in the context of the family. As individuals become integrated into UK society, they may therefore reject existing representations, adopt the dominant social representation or use hybrid representations as seen in the example of the Chinese communities in the UK (Gervais and Jovchelovitch, 1998). Similarly, it is possible that like Jodelet’s French villagers, the UK public use co-existing and at times conflicting representations depending on the demands of the situation. There is no research on the social representations of people living with V.I. held by the UK public. Considering the wider scope of social representations, this would be a useful exploration to better understand how the two groups relate to each other. Research from Greece found that multiple lay understandings of prejudice and racism co-existed in this sample, however these were rarely used to explain discrimination and hostile behaviour (Figgou and Condor, 2006). Instead, participants associated discriminatory behaviour with fear and insecurity rather than racism (Figgou and Condor, 2006). While Figgou and Condor’s (2006) participants distanced themselves from racism and prejudice, evoking fear and insecurity may serve to excuse behaviour or lack of behaviour. Although they also expressed openness and welcomed the opportunity to make a new friend, the 42 UK adults who were asked how they would feel about sitting next to a person who was blind at a dinner party, described feelings of anxiety, confusion and awkwardness about what to say and do, not wishing to offend and ensuring the person with V.I. was included (Versiti, 2018). In some cases, there may then be a danger that fear and insecurity may excuse people from helping their peers with V.I. However, the role of fear in preventing behaviour will need to be explored in future research.

### 6.3 Limitations

A number of constraints on the generalisability of findings need to be noted. First, there was a paucity of research on the topic resulting in an undue focus on a small number of studies (e.g., Grewal et al., 2002; Moss and Frounks, 2022) which explored a wider spectrum of attitudes, perceptions and behaviours. Second, the majority of research reviewed in the preceding sections has been commissioned by charities in the disability or V.I. sector and conducted by a small number of market or social research agencies. This may reflect the need for research agencies with capacity to recruit and collect data from large samples to explore public attitudes. In some cases, this gives findings for a nationally representative sample, although people who do not speak English, do not live in the community (e.g., those who live in care homes) and/or do not have capacity to give informed consent tend to be excluded from research. Moreover, most findings have been published in charity or government reports and have therefore not undergone peer-review. Most of the research is observational in nature and uses self-report (which is subjective to various recall and social desirability biases) rather than objective measures, and questions which were designed for the research rather than validated scales to assess attitudes, perceptions and behaviours. For instance, Nikolaraizi and De Reybekiel (2001) adapted an existing attitudes scale for their research but did not validate it. Similarly, for BSA, existing questions are used or adapted where possible. These are tested in a small-scale pilot and go on to form part of a time series. Moreover, BSA includes a nationally representative sample of adults living in Great Britain, which excludes Northern Ireland. Finally, there was a lack of conceptual clarity in the research, particularly relating to the distinction between attitudes and perceptions. As such, findings relating to attitudes, behaviours and perceptions tended to be presented together without distinguishing between the three. For instance, willingness to sit next to a child with V.I. (Nikolaraizi and De Reybekiel, 2001) is arguably an indication of intended behaviour rather than an attitude. This made it difficult to disaggregate findings for these two concepts in the current article.

## 7 Conclusion

This narrative review provides an overview of public attitudes, perceptions and, by extension, behaviours towards people living with V.I., with a focus on the UK context. Beyond this, it explores how social psychological theories can deepen our understanding of the reported behaviours, attitudes and perceptions and mismatches between these. As such the article provides an example how existing social psychological theories may serve to address issues in an applied context. Despite their potentially adverse impact on identity, health and well-being, relatively little research has explored these topics from the perspective of the UK public. Most of the available research is observational in nature and relies on self-report rather than objective measures. Moreover, the focus has largely been on assessing explicit attitudes usually towards people with disability in general rather than V.I. specifically. More research is required to understand the reason for the mismatch between the negative experiences reported by people with V.I. and the relatively positive attitudes among UK adults before work can begin on developing interventions to improve intergroup contact experiences.

## Author contributions

NH: Conceptualization, Data curation, Investigation, Methodology, Project administration, Writing – original draft, Writing – review & editing. LJ: Writing – review & editing, Writing – original draft, Validation. FB: Writing – review & editing, Writing – original draft. ES: Writing – review & editing, Writing – original draft. RG: Funding acquisition, Resources, Writing – original draft, Writing – review & editing.

## Footnotes

^1^This may reflect the lower proportion who consider an older person with a hearing aid to be disabled (44%). In contrast, 91% considered a person in a wheelchair to be disabled.

^2^Not all participants were asked all questions. Subsample sizes for individual questions may therefore be lower.
